# Aptamer-Based Point-of-Care Devices: Emerging Technologies and Integration of Computational Methods

**DOI:** 10.3390/bios13050569

**Published:** 2023-05-22

**Authors:** Yusuf Aslan, Maryam Atabay, Hussain Kawsar Chowdhury, Ilgım Göktürk, Yeşeren Saylan, Fatih Inci

**Affiliations:** 1 UNAM—National Nanotechnology Research Center, Bilkent University, Ankara 06800, Turkey; 2 Institute of Materials Science and Nanotechnology, Bilkent University, Ankara 06800, Turkey; 3 Department of Chemistry, Hacettepe University, Ankara 06800, Turkey

**Keywords:** aptamer, aptasensor, biosensor, computational methods, nanomaterials, point-of-care

## Abstract

Recent innovations in point-of-care (POC) diagnostic technologies have paved a critical road for the improved application of biomedicine through the deployment of accurate and affordable programs into resource-scarce settings. The utilization of antibodies as a bio-recognition element in POC devices is currently limited due to obstacles associated with cost and production, impeding its widespread adoption. One promising alternative, on the other hand, is aptamer integration, i.e., short sequences of single-stranded DNA and RNA structures. The advantageous properties of these molecules are as follows: small molecular size, amenability to chemical modification, low- or nonimmunogenic characteristics, and their reproducibility within a short generation time. The utilization of these aforementioned features is critical in developing sensitive and portable POC systems. Furthermore, the deficiencies related to past experimental efforts to improve biosensor schematics, including the design of biorecognition elements, can be tackled with the integration of computational tools. These complementary tools enable the prediction of the reliability and functionality of the molecular structure of aptamers. In this review, we have overviewed the usage of aptamers in the development of novel and portable POC devices, in addition to highlighting the insights that simulations and other computational methods can provide into the use of aptamer modeling for POC integration.

## 1. Introduction

The growing number of discoveries on the chemical pathways taken by diseases have driven our recent advancements in medicine and biomedical technologies [1,2,3]. Tracking biomarkers in our bodies provides us with plentiful information about a number of health conditions, such as an indication of specific physiological conditions, the presence of a disease, the progress of treatment, and the risk of disease development. The presence of certain biomarkers can be recognized as a conformation of diseases, such as cancer [4], cardiovascular diseases [5], neurodegenerative diseases [6], viral infections [7], and organ injuries [8]. Therefore, the application of in vitro biomarker detection has been expanded to many diseases; accordingly, the necessity of rapid and cost-effective solutions has increased in our search for the early diagnosis of life-threatening diseases.

Today’s widely employed conventional biomarker quantification tools include mass spectrometry [9], cell culture, polymerase chain reaction (PCR), enzyme-linked immunosorbent assay (ELISA), Western blot, and flow cytometry [10]; however, these methods are difficult to integrate into financially-restricted and resource-scarce settings. Metamorphosing the current conventional practices with point-of-care (POC) systems is vital to delivering healthcare to patients in developing countries more efficiently. Therefore, the qualifications of ideal POC devices have been standardized with a set of criteria known as ASSURED, which stands for affordability, sensitivity, specificity, user-friendliness, rapid, equipment-free, and deliverable [11,12]. Currently, ASSURED is evolving into a newly suggested acronym called RE-ASSURED [13]. The addition of real-time connectivity, ease-of-specimen collection, and environmentally friendly qualifications are justified by the rapid transmission of data to individuals/patients; the increased feedback availability for treatment monitoring, possibly over smartphones or other such mobile systems; the provision of noninvasive sample analysis, with an increased capacity for self-testing; and a reduction of the risks related to environmental hazards [13]. Concentrating this focus on limited resources and expanding POC technologies into multiple settings for self-testing purposes, the ideal POC device requires many qualifications, such as low cost, portability, short turnaround time, specificity, and sensitivity at the first glance. Conventionally, antibodies have been utilized as the recognition elements in POC systems in order to detect markers in biospecimens. However, in some cases, high specificity over many targets can be impeded due to single-point mutations or conformational isomers [14]. This being the case, aptamers shine out as a strong candidate for replacing antibodies since they are tertiary structures of relatively short nucleic acid sequences that can selectively recognize the desired target with high affinity and specificity, as well as having the capacity to be easily customized for such changes [15]. The complementary base pairs found in aptamers tend to form secondary structures (stem ring, hairpin, spiral, pseudoknot, clover, and so on), which can collectively combine into tertiary structures [16]. These structures can be computationally predicted by the identification of Watson–Crick base pairing regions [17]. The properties of aptamers have been explained in more detail in the following section. On the other hand, the utilization of simulations and other such computational methods (e.g., Molecular Docking calculations, Molecular Dynamics (MD) simulations, Density Functional Theory (DFT), Quantum Mechanics and Molecular Mechanics (QMMM), and Artificial intelligence (AI) methods) to design a biosensing system for POC applications has not been encountered frequently. Combining and validating these studies with experiments would hold significant potential for aiding scientists and individuals as end-users by predicting experimental drawbacks, thus reducing the number of false interpretations, shortening the optimization time, and reducing both unnecessary material usage and the associated costs. Therefore, the impact of simulations and computational methods in POC applications is often highlighted.

In this study, we have reviewed both the significant benefits of aptamers and the current emerging applications for aptamer-oriented POC diagnostic platforms, in addition to discussing the integration of simulation and computational methods.

## 2. Aptamers

The idea for this use of single-stranded oligonucleotides (aptamers), ribonucleic acid (RNA), or single-strand deoxyribonucleic acid (ssDNA) was initially proposed in 1990 [18]. Although RNA and ssDNA aptamers can have different sequences and folding patterns, they can be designed to bind to the same target [19]. These single-stranded oligonucleotides have unique tertiary structures, which allow specific interactions with target molecules. Aptamers are generated in vitro through the Systematic Evolution of Ligands by Exponential Enrichment (SELEX) approach [20,21], which can be conducted against different molecules, including proteins, small compounds, cells, and nanoparticles [18]. The SELEX method involves repeated rounds of selecting and amplifying target-specific nucleic acid sequences from a large pool of random sequences. The conventional SELEX approach includes the combination of a random nucleic acid library with the target molecules, the removal of non-target-specific sequences, the amplification of target-specific sequences, and the characterization of the isolated aptamers [22,23]. The whole process is repeated, once the selected aptamers are sufficiently specific to the target and demonstrate the intended levels of the enrichment. Over the last three decades, researchers have made various updates to the traditional SELEX strategy in order to boost its efficiency and cost-effectiveness [24] while, at the same time, increasing the selectivity and affinity of the generated aptamers [20]. Such advances included streamlining the selection procedure, reducing generation time, and optimizing the amplification and characterization of target-specific aptamers. Immunoprecipitation-coupled SELEX, for instance, was developed to enhance affinity under normal physiological scenarios [25], whereas the capture-SELEX was established to improve the selection effectiveness of aptamers for unidentified small molecular targets [26]. Furthermore, whole cells [27], tissue or organs in animals [28] have also been utilized as SELEX tools for distinguishing between healthy and diseased conditions [29], monitoring prognosis [30], and discovering new biomarkers [31]. Emergent technologies, such as microfluidics [32], capillary electrophoresis [33], atomic force microscopy [34], and several in silico approaches, have also been used to improve the performance at the current stage of SELEX strategy.

**Figure 1:**
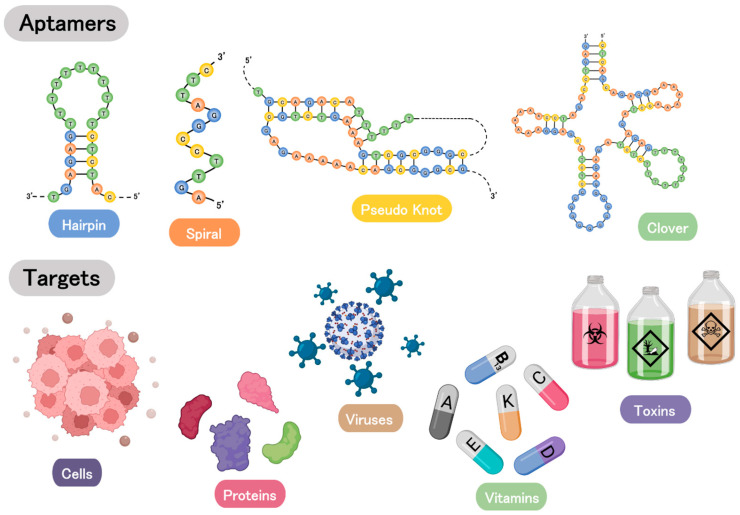
The structures and targets of aptamers. Created with BioRender.com (accessed on 13 March 2023).

As shown in Figure 1, hairpin, spiral, stem ring, pseudoknot, and clover are common structures of SELEX-generated aptamers that can bind to many targets, such as, proteins, toxins, viruses, and vitamins [35,36]. These aptamers have been studied for their applications in diagnostic and therapeutic purposes [19]. Owing to their high affinity and other properties exclusive to aptamers toward the target proteins, they have been employed as a biorecognition element in biosensors as an alternative to antibodies [37]. In addition, in comparison with antibodies, aptamers have many favorable advantages: (1) They are chemically and structurally stable. While antibodies easily undergo irreversible denaturation, aptamers can recover their native conformation by tuning the conditions, such as changing the pH of the medium (slight changes), salt concentration, chelating agents, and temperature. Aptamers are not damaged in many of these conditions, except for the extremes of pH value, wherein the structure of the aptamer can be irreversibly damaged. (2) Furthermore, the production of monoclonal antibodies is laborious and very expensive, while aptamers can be synthesized in greater quantity, accuracy, and reproducibility, and at a relatively lower cost. (3) In addition, aptamers can be chemically modified or conjugated without sacrificing their binding affinity, whereas antibodies require stochastic modifications, which bring a possible loss in binding activity [14]. (4) They can be stored for a long time without losing their activities because of thermal denaturation [38,39,40,41]. (5) Lastly, aptamers have no or low toxicity and immunogenicity, which is crucial for in vitro and in vivo applications [42,43]. Aptamers are good candidates for biosensors that use optical, electrochemical, and mass-based detection techniques, and are compatible with antibodies. In addition, they have been studied for the creation of biosensors with high detection, sensitivity, and stability [44]. Despite the advances in SELEX procedures, along with all the properties and applications of the aptamers that have been considered, studies show that some aptamers are unable to bind the target molecule [45]. As an example, Zong and Liu showed that there was no specific binding in the structure of arsenic(III)-binding aptamer, despite this structure having been used and reported on in two dozen peer-reviewed articles, previously [46]. There are some new analytical techniques for a more in-depth understanding of the aptamer-target structures. In these techniques, (a) the kinetic and thermodynamic information of the binding event and (b) the structure of aptamer and aptamer-target complex are investigated. In part (a), analytical methods are divided into two groups: those methods in which interactions are studied in solution, and those methods in which aptamer-target kinetics are characterized while one of the biding structures is immobilized. Nowadays, aptamer immobilization protocols are used for designing electrochemical, mass-sensitive, or optical aptasensors. Part (b) is subdivided into two types of techniques: high-resolution methods, such as nuclear magnetic resonance (NMR) spectroscopy, X-ray crystallography, and electron microscopy (EM); and low-resolution techniques. Low-resolution techniques that can provide information about the size and shape of the aptamer include small-angle X-ray scattering (SAXS) and circular dichroism (CD) spectroscopy [45]. 

In addition to the simulation and computation methods that were briefly mentioned in the previous section, one novel (and unconventional) way to select aptamers with a high affinity for targeting molecules is to perform Artificial intelligence (AI)-assisted strategies. AI, including machine/deep-learning algorithms, is a strong and accurate approach for predicting the interaction between aptamers and targets. Therefore, the combination of the simulation and computation methods (which are structure-based methods) with AI methods could be a promising approach to predicting interactions between aptamers and target molecules [47]. 

In the next sections, different types of aptasensors will be discussed in detail, along with their applications. 

## 3. Aptasensors in POC-Based Biosensing Platforms

Immunochromatography tests are largely employed for monitoring infections, diseases, and other health conditions in POC settings [48,49]. The biomolecules used as probes in the POC devices have included glycan, aptamers, enzymes, nucleic acids, and antibodies [48,49,50,51]. The vast majority of targets that can be captured by aptamers are small metal ions and whole cells. With the help of aptamers, medical devices that utilize aptamer-coupled POC functions bring their capabilities from the lab to the bed-side, and thus play a significant role in the healthcare sector. Aptamer-based biosensors are useful for many diagnostic procedures, including the detection of illness, cancer, heart disease, etc. Current techniques for illness diagnosis with POC have been developed using a simple lateral flow system, or modest electrical and optical-based systems [48,51,52]. Furthermore, the integration of paper, nanomaterials, portable, and smartphone POC-oriented aptamer-based biosensors (aptasensors) can be divided into various platforms according to the type of transducing mechanisms: colorimetric, fluorescent, surface plasmon resonance (SPR), and electrochemical (Table 1). When evaluated on these sensing devices, aptamers made against various biomarkers showed a variety of detection limit ranges. In the following sections, these aptasensor strategies will be discussed in detail.

### 3.1. Colorimetric Aptasensors

Colorimetric methods provide visual recognition for aptamer-target binding, which is either discerned with the naked eye or measured quantitatively using an optical reader. The concentration of the detected targets is determined by any alterations in scattering and absorption efficiencies [94], the refractive index of the surrounding medium [94], the electromagnetic spectrum [95] or wavelength, and the full width at half maximum (FWHM) values of the resonance signal [96]. Such methods hold great potential for on-site diagnosis, especially in resource-limited settings, since they are cost-effective and straightforward [97]. However, the laborious fabrication process [98], inefficiency in detecting multiple targets [99] and narrow working pH range are some of the current obstacles in colorimetric aptasensors [100]. Herein, we have classified this concept into two subcategories according to their fabrication substrates, i.e., paper-based and ‘in solution’ colorimetric aptamer assays.

#### 3.1.1. Paper-Based Colorimetric Aptasensors

Paper is an excellent and cost-effective off-the-shelf material for immobilizing biomolecules (e.g., protein, nucleic acids, aptamers, antibodies, and so on) [101,102,103]. Assays on paper require minimal sample volume and provide quick visual results without external instruments. These assays can also be stored in ambient conditions with temperature-stable modified aptamers [16]. Although paper-based biosensors have many pros, as stated above, they also come with some limitations, including: (i) sample evaporation or entrapment on the paper substrate; (ii) instability, due to environmental interference factors; (iii) reader requirements for high sensitivity and quantitative results; and (iv) low mechanical stability [104]. Additionally, false negative and false positive outcomes would be possible for these platforms, due to concentrations of the target so low as to fall below the LOD value [105], or from the cross-reactivity between structurally similar targets [106], respectively.

Lateral flow assay (LFA) is a common paper-based method, allowing for on-site biomarker detection within minutes, and using naked-eye or portable systems. LFAs use two essential sensing strategies: (1) sandwich, and (2) competitive assays. Sandwich assays capture targets between detection and capture bioreceptors for selective signal generation [107], whereas competitive assays use target competition to detect either labelled bioreceptors [108] or capture bioreceptors [109].

**Figure 2:**
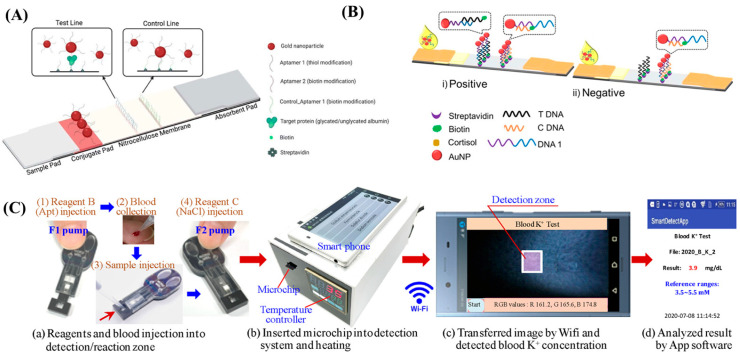
Examples of paper-based POC aptasensors: (**A**) Sandwich LFA using streptavidin-biotin immobilization for simultaneous capturing albumin and glycated albumin on test line [55]; (**B**) Working principle of cortisol LFA with an adsorption–desorption sensing mechanism, where either (i) cortisol capture is detected via AuNPs-linker hybridization on the test line (positive result), or (ii) no cortisol is captured on the test line (negative result) [53]; (**C**) operations steps of paper-based microfluidic integrated blood potassium concentration detection device [58].

For an example of the sandwich format, an LFA strategy tracked both glycated albumin and albumin concentrations in serum to record the glycemic status of patients with gestational diabetes mellitus (GDM), a glucose intolerance disorder in pregnant people [55]. The assay was able to detect albumin as a target molecule between AuNPs conjugated with both detection aptamer and capture aptamer on the test line. The positive result was discerned from the appearance of red bands on both the test line and control line. The dual assay also used a smartphone-integrated handheld colorimetric reader for quantitatively tracking albumin status in serum. Furthermore, the platform detected glycated albumin and serum albumin concentrations as low as 0.8 mg·mL^−1^ and 1.5 mg·mL^−1^, respectively (Figure 2A). Another study simultaneously monitored platelet-derived growth factor-BB (PDGF-BB) and thrombin, which are biomarkers for several tumor regions (e.g., liver and gastrointestinal tract) and for coagulation abnormalities, respectively [56]. There were two different test lines and a single control line present on the assay. AuNPs-labelled PDGF-BB specific aptamers and AuNPs-labelled thrombin specific aptamers were utilized as conjugated detection probes. In the presence of targets, the detection probes were separately captured in their respective test lines. The third band was used as a control line for capturing excess detection probes. A portable colorimetric reader was also used for determining the target concentrations. This assay provided LOD values of 1.0 nM and 1.5 nM for PDGF-BB and thrombin, respectively. By way of contrast, a competitive assay has been reported for the detection of CA125, an ovarian cancer serum biomarker, in serum samples [110]. The assay utilized the intrinsic peroxidase activity of AuNPs for catalyzing the oxidation reaction of DAB/H_2_O_2_ substrate, where the excess amount of AuNPs would result in an enhanced color intensity, and a deficiency of AuNPs would lead to a lower color intensity. During the assay operation, a consistent amount of AuNPs-labelled CA125 biomarkers were placed on the conjugate pad, where they competed with CA125 in the sample to bond with the immobilized CA125 specific aptamers present on the test line. Therefore, the concentration of the bound CA125 was inversely proportional to the color intensity on the test line. Further, the concentrations of biotargets were determined with grayscale intensities of test line images captured on a smartphone. The assay was able to detect CA125 down to 5.21 U·mL^−1^ within 20 min.

In addition, some studies were designed to combine antibodies and aptamers in a single hybrid LFA. In such an example, CXC-motif chemokine ligand (CXCL) 9 (a critical urinal biomarker for antibody-mediated rejection of kidney transplantation) was detected on a tracking sandwich format LFA [57]. Antibodies specific to CXCL 9 were initially utilized as capture probes on the test line, and the detection aptamers specific to CXCL 9 were conjugated to AuNPs to ensure signal transduction. The usage of aptamer and antibody in combination improved the binding efficiency of CXCL 9. Further, the capture aptamers specific to the AuNP-conjugated detection aptamers were also immobilized on the control line to secure the correct operation of the assay. In the presence of CXCL 9 in urine samples, two different red bands were discerned for designating a positive result. Furthermore, the LOD of the assay was reported as 10 pg·mL^−1^, and the specificity of the assay was determined to be 71% by comparing the estimated glomerular filtration rates (eGFR) of patients, which is a blood test parameter for renal function [111].

In addition to sandwich and competitive format assays, LFAs also exploited the target-induced conformational change in the labelled aptamers through adsorption–desorption mechanisms [112,113,114]. The working principle of this strategy relies on the adsorption of an aptamer on the surface of a label, and the following desorption of the aptamer from the label after their conformational changes upon interaction with the target molecules [112]. In the literature, this sensing strategy has also been combined with duplex aptamers, where the labelled duplex aptamers were disassociated from their complementary aptamers via target-aptamer binding. An example of this strategy was demonstrated for monitoring cortisol—a stress level-associated steroid hormone—in human saliva [53]. In this assay, duplex DNA was formed with the hybridization of AuNPs-conjugated linker DNA and cortisol-specific DNA aptamer. The duplex DNA aptamer was separated from its complementary aptamer in the presence of cortisol due to the conformational change in the aptamer, and the cortisol-bound aptamer was released. The remaining linker-AuNP conjugation was captured using a linker complementary strand on the test line. Furthermore, the assay was validated with a different complementary sequence on the control line which was able to interact with both separated and unseparated duplex DNA structures (Figure 2B). Cortisol was detected with a LOD of 0.37 ng·mL^−1^ with the use of this assay. The same research group have also employed a similar duplex DNA dissociation strategy for dopamine detection in urine, which was able to detect dopamine concentration as low as 10 ng·mL^−1^ [54].

The integration of microfluidic platforms with disposable paper strips has also been utilized for aptamer-based biomarker detection applications [115,116,117,118,119,120,121]. Recently, a low-cost and smartphone-adapted sensing platform was introduced for measuring potassium ion (K^+^) levels in whole blood, which is an essential parameter for chronic kidney disease [58]. In short, the platform comprises PMMA-sealed paper microfluidic chip and finger pumps for transporting different reagents into the reaction chamber. A four-strand aptamer structure was utilized as a detection probe, which conformationally changed into a G-quartet structure upon interaction with K^+^ ions. At first, the aptamers were adsorbed onto the surface of the AuNPs via electrostatic force. Then, whole blood was introduced to the AuNPs–aptamer complex, and the complex conformationally turned into G-quartet structures after target interaction, releasing the AuNPs due to a weakened electrostatic force. Finally, the bare AuNPs were aggregated with NaCl introduction, and transduced a K^+^ ion-specific color change (Figure 2C). The LOD of the platform was reported as 0.01 mM, and had an affordable fabrication cost (US $0.50 per microchip).

#### 3.1.2. In-Solution Colorimetric Aptasensors

**Figure 3:**
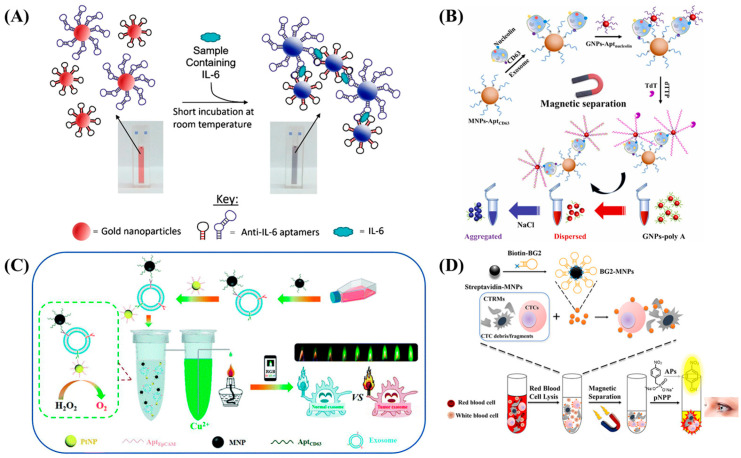
Working principle schematics of solution-based POC aptasensors: (**A**) The aggregation of AuNPs-integrated complementary sandwich aptamers for IL-6 determination [62], (**B**) Integration of MNPs and AuNPs to aptamers for the tracking of tumor-expressed exosomes [61], (**C**) Visual detection of magnetically isolated exosomes via Cu^2+^ driven atomic flame assay [133], (**D**) Isolation and tracking of CTCs via MNPs driven magnetic separation and AP catalyzed enzymatic visual assay, respectively [136].

Though in-solution colorimetric aptasensors are rapid and easy to operate, they require a relatively large reagent volume than those of paper-based platforms [122]. They mostly utilize nanoparticles or enzymes to produce a color change, relying on the concentration of target analytes. In addition, metal nanoparticles, especially AuNPs, have an intrinsic localized surface plasmon (LSPR) property, where the electrons are collectively oscillating between a nanostructure and dielectric interface that is induced with the electromagnetic interaction of incident light beam [7,123,124,125]. This phenomenon has benefited numerous colorimetric sensing platforms since the absorption spectrum of these materials was found in the visible region of the electromagnetic spectrum [126]. Many solution-based aptasensors utilize the absorption–desorption mechanism of aptamers onto AuNPs due to the changes in electrostatic interactions. A common working principle is that aptamer functionalized AuNPs are protected against NaCl until they are subjected to a conformational change upon target binding. Aptamers are released from the surface of AuNPs due to target–aptamer binding. Desorption of aptamers weakens the electrostatic repulsion between AuNPs and exposes the particles to negative charge neutralization (NaCl). This leads to AuNPs aggregation which causes a color change from red to blue [127]. An example of such a sensing mechanism was reported for monitoring the levels of human epidermal growth factor receptor 2 (HER2), a potential breast cancer biomarker, in human serum [59]. HER2 specific aptamers were initially adsorbed on the surface of AuNPs after an incubation step. Upon HER2 introduction into AuNPs–aptamer structures, the AuNPs were released due to the HER2 binding induced conformational changes. Further, NaCl was applied to the solution for aggregating bare AuNPs, leading to a HER2 concentration-dependent color change. This assay provided a rapid and cost-effective platform for breast cancer diagnosis with a LOD of 10 nM. A similar AuNPs aggregation strategy was introduced for capturing retinol-binding protein 4 (RBP4), a potential biomarker for the early diagnosis of type 2 diabetes mellitus [60]. This assay improved some contents and assets of conventional RBP4 detection methods, such as ELISA and Western Blot, and reduced both the response time (from several hours to 5 min) and LOD values (90.76 ± 2.81 nM). Another study demonstrated the detection of interleukin-6 (IL-6), a peptide used for the early diagnosis of brain injury or inflammation, in a bed-side setting [62]. In this study, a sandwich type of two complimentary aptamer-AuNPs conjugates were employed for the capture of IL-6 where each aptamer targeted a different binding location of IL-6. The color of AuNPs changed from red to pink within 5 min of introducing the IL-6 (Figure 3A). The assay was able to detect IL-6 down to 1.95 μg·mL^−1^ within a concentration range of 3.3–125 μg·mL^−1^.

In addition to protein biomarkers, researchers have performed significant research on the detection of extracellular vesicles, which carry crucial information in a packaged nano- to micron-sized entities [128,129,130,131]. As an example, a platform was developed for the detection of leukemia-derived exosomes using terminal deoxynucleotidyl transferase and salt-induced AuNPs aggregation. Magnetic beads were used to isolate exosomes, which were selectively captured using nucleolin–aptamer AuNPs (Figure 3B). Thereafter, the LOD was improved to 42 particles·μL^−1^ using a double signal amplification method [61]. Despite most of the in-solution studies using a direct visual output with salt-induced AuNPs aggregation, one study utilized the photothermal effect of AuNPs for tracking biomarkers via temperature change. In this study, the photothermal effect of AuNPs was used for tracking biomarkers via temperature changes for a DNA detection assay targeting Mycobacterium tuberculosis. AuNPs that had been conjugated with ssDNA interacted with target DNA, leading to the aggregations, which had a high photothermal effect under a near-infrared laser. The LOD of the assay was 0.28 nM, with a response time under 40 min [63]. Metal nanoparticles rather than AuNPs were also indirectly employed for provoking a target-specific color intensity [132]. Such a study was reported for tumor-originated exosome detection by indirectly exploiting platinum nanoparticles (PtNPs) [133]. Researchers used PtNPs to reduce H_2_O_2_ into O_2_, generating a flame emission that correlated with captured CTC-originated exosomes. Exosomes were sandwiched between CD63-specific aptamer-functionalized MNPs and EpCAM-modified PtNPs (Figure 3C). The number of exosomes was measured using the changes in the green intensity of the atomic flame, with a LOD of 7.6 × 10^2^ particles·mL^−1^ within a 1 min reaction response. Silver nanoparticles (AgNPs) have also gathered attention as a colorimetric label for aptamers due to their availability as a cost-effective material, and their ease of functionalization [134]. One study developed a simple colorimetric detection method for adenosine in human urine using AgNPs and adenosine-specific aptamers [135]. The aptamers prevented particle aggregation in high NaCl concentrations and complexed with adenosine to stabilize the particles against aggregation. The concentration of adenosine was determined by measuring the dispersity and absorption intensity of the AgNPs, with a LOD of 21 nM in a linear range of 60–280 nM. Beyond nanoparticle-induced color change, enzymes were also utilized for colorimetric aptasensors. Such an example was reported for the capture of alkaline phosphate (AP) isozymes (placental alkaline phosphatase (PLAP) and intestinal alkaline phosphatase (IAP)) for the clinical diagnosis and prognosis of colorectal cancer. PLAP–IAP isozymes were initially captured using MNPs functionalized aptamers, which had affinity against AP heterodimers. Target isolation was obtained with the magnetic separation of aptamer-MNPs. PLAP–IAP isozymes catalyzed a chromogen (p-nitrophenyl phosphate, pNPP) using an enzyme–signal amplification mechanism for inducing a color change in the presence of the target (Figure 3D). The receiver operating characteristic (ROC) analysis of the assay demonstrated a sensitivity of 92% and the specificity as 82% compared to the results derived from the ones in clinical practice [136]. The emergence of novel nanozymes, i.e., artificial nanomaterials that exhibit physicochemical properties of natural enzymes (e.g., Fe_3_O_4_ nanoparticles), has presented attractive application alternatives to conventional catalytic labels (e.g., horseradish peroxidase: HRP) [137]. A nanoenzyme-based sandwich formatted aptamer sorbent assay (NLASA), for instance, was reported for monitoring platelet-derived growth factor-BB (PDGF-BB) using naked eye detection [64]. The assay operated similarly to a regular sandwich-based ELISA where, instead of the antibody–enzyme complex, aptamer-functionalized Fe_3_O_4_@C nanowires were utilized as nanozymes for catalyzing the oxidation reaction of tetramethylbenzidine. This reaction caused a color change in the presence of PDGF-BB. The usage of single dimension nanowires eliminated the drawbacks caused by the instability of regular peroxidase enzymes. This aptasensor was able to detect down to 10 fM, within a working range between 10 fM to 100 fM.

### 3.2. Fluorescent Aptasensors

Fluorescent aptasensors are capable of high sensitivity (low signal-to-noise ratio) with a rapid turnaround time [138]. The generation of fluorescence response is obtained using the modification of aptamers with fluorophores, fluorescent proteins, quantum dots (QDs), and upconversion nanoparticles (UCNPs) [139]. Target induced-conformational change of fluorescently labelled aptamers provides the opportunity for developing fluorescence quenching or fluorescence enhancement coupled aptasensors. Therefore, we have classified fluorescent aptasensors into two subcategories according to their sensing mechanisms, i.e., fluorescence quenching or metal-enhanced fluorescence (MEF or plasmon-enhanced fluorescence (PEF)).

#### 3.2.1. Fluorescence Quenching-Based Aptasensors

Förster resonance energy transfer (FRET) is a nonradiative energy transfer between donor and quencher, useful for monitoring biorecognition changes [140,141]. The efficiency of FRET depends on the spectral overlap, separation distance, and relative orientation [142,143,144]. By modifying aptamers with quenchers and fluorophores, fluorescence quenching can be used as a tool for biomarker tracking by observing the aptamer conformational dynamics.

**Figure 4:**
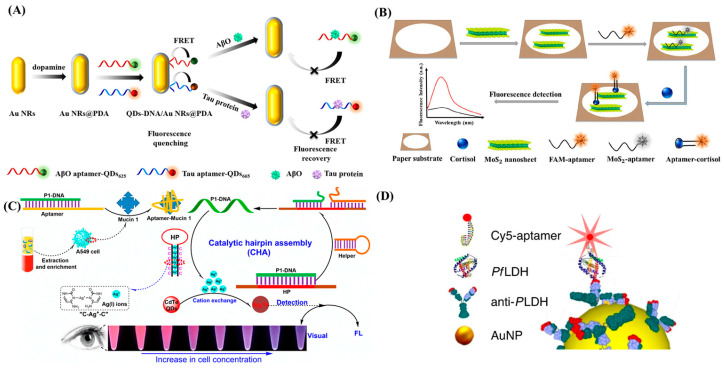
Fluorescence biosensors for aptamer integrating POC: (**A**) FRET assay based on QDs modified ssDNA utilization on polydopamine coated AuNRs for the early diagnosis of Alzheimer [66], (**B**) paper-based and MoS_2_ induced fluorescent quenching assay for cortisol monitoring from sweat samples [70], (**C**) CHA amplified and fluorescence quenched assay for visual detection of CTCs [67], and (**D**) a hybrid antibody and aptamer assay for MEF mediated malaria tracking [72].

Recently, steroid hormones have been observed to quench fluorescence signals of QDs by means of surface adsorption, which was utilized to develop a selective cortisol (a biomarker for numerous diseases including Cushing’s syndrome and stress disorders) monitoring assay in saliva [65]. QDs that had been functionalized with MNPs were modified with cortisol-specific aptamers for fluorescence quenching. The assay exhibited an LOD down to 1 nM within a 20 min turnaround time. FRET was used for simultaneous detection of the Alzheimer biomarkers Aβ and tau protein with polydopamine-capped gold nanorods (AuNRs@PDA) and dual-color CdSe/CdS/ZnS QDs [66]. Aptamer-target binding caused conformational changes and selective fluorescence recovery under single wavelength excitation (Figure 4A). The aptasensor had LOD values of 50 pM for Aβ and 10 pM for tau, providing a simple and effective Alzheimer diagnosis platform compared to ELISA. A wearable and paper-based FRET aptasensor was developed for monitoring cortisol concentrations [70]. MoS_2_ nanosheets on carboxyfluorescein-modified aptamers formed the FRET mechanism. The hybrid 3D origami microfluidic device integrated with a custom build smartphone-mounted detector was used to monitor fluorescence recovery (Figure 4B). The presence of cortisol in the perspired sweat intervened with the FRET mechanism by conformationally changing the aptamer structure. The fluorescence intensity was recovered proportionally to the captured amount of cortisol, and the LOD was 6.76 ng·mL^−1^ in artificial sweat.

Apart from fluorescent dyes, UCNPs have gained attention for their reduced interference of autofluorescence of paper substrates and scattering lights from biological samples [145] due to their ability to convert multiple low-energy photons into a single high-energy photon through NIR excitation [146]. For instance, UCNP–aptamer–TAMRA was employed to detect IgE, an allergic biomarker, on a paper-based fluorescent assay [71]. The aptamer’s stem–loop structure caused a luminescence energy transfer, a type of nonradiative energy transfer phenomenon similar to the FRET mechanism. The addition of IgE disrupted the structure and increased the distance between the UCNPs and the TAMRA, recovering a fluorescence signal proportional to the captured IgE, with a LOD of 0.13 IU·mL^−1^. A study showed the detection of mucin 1 (a surface protein of circulating tumor cells (CTCs)) through simultaneous visual and fluorescent responses [67]. Catalytic hairpin assembly (CHA) and cation exchange reactions were combined for the transduction of signals (Figure 4C). The mucin 1-aptamer underwent a conformational change in the presence of mucin 1, and then, accordingly, released P1-DNA, which was hybridized with Ag+-functionalized hairpin DNA. The released Ag+ ions bound to QDs, quenching their fluorescence and transducing a visible color change proportional to the amount of mucin 1 captured. The LOD was reported as 0.15 fg·mL^−1^ mucin 1 or 3 CTCs·mL^−1^. A hybrid biosensor using both antibodies and aptamers was developed to detect alpha-fetoprotein (AFP), a biomarker for hepatocellular carcinoma [68]. The biosensor utilized silica-coated CdTe QDs, in addition to anti-AFP monoclonal antibodies conjugated to AuNPs, with the sandwich structure bringing the AuNPs and QDs in close proximity for FRET. The LOD was reported as 400 pg·mL^−1^, with a dynamic range of 0.5–45 ng·mL^−1^. Fluorescence quenching was used in a hollow hydrogel microneedle biosensor for detecting glucose, ATP, L-tyrosinemia, and thrombin in interstitial fluid [73]. Aptamers conjugated to a fluorophore and a quencher functionalized DNA competitor were used. The hybridization was disrupted in the presence of targets, causing the fluorescence signal to be recovered. The assay had a 2 min turnaround time, and the LOD values were reported as 1.1 mM, 0.1 mM, 3.5 µM, and 25 nM for glucose, ATP, L-tyrosinamide, and thrombin, respectively. The performance of this biosensor could be improved with different fluorophores for each aptamer probe to increase target specificity.

#### 3.2.2. Metal-Enhanced Fluorescence-Based Aptasensors

The fluorescence signal of low-concentration biomarkers requires bulky instruments for efficient measurements, thereby limiting their expansion into the POC and bed-side settings [147]. There are many efforts to resolve such challenges through the introduction of metal-enhanced fluorescence (MEF) via metal/metal oxide nanostructures (e.g., nanoparticles, nanorods, nanocavities, nanoholes, planar surfaces, etc.) [147,148,149]. In short, MEF is the enhancement of fluorescence due to the coupling effect between fluorescence excitation/emission and plasmonic resonance of surface plasmons confined to metal nanostructures [147,150]. For an efficient MEF application, the distance between metal nanostructures needs to be optimized (mostly between 20–50 nm for planar plasmonic structures [147]), since nonradiative quenching effects (such as FRET) may dominate at short distances (mostly between 1–10 nm [151]). Recently, aptamers are shown to be performed on MEF biosensors due to the tunability of surface plasmon–fluorophore separation distances with the conformational change in the aptamers [152]. An example of a solution-based MEF strategy was tested in detecting mutated breast cancer gene-1 (BRCA-1), a pathogenic variant of tumor suppressor genes [153], by utilizing FRET and MEF strategies at the same platform [69]. Two AuNPs, different in size (60 nm and 20 nm, respectively), were modified by combining dsDNA and ssDNA. Clustered regularly interspaced short palindromic repeats (CRISPR) is an evolving gene editing tool that recognizes and cleaves target nucleic acid sequences with the use of the Cas 12 protein, which sweeps around the nucleic acid sequence and determines the target for cleavage. In the study, two AuNPs were combined by modifying them with ssDNA and fluorescein isothiocyanate (FITC) functionalized dsDNA. This interaction formed FRET, since the particle distance between FITC and AuNPs was 2 nm only. In the presence of BRCA-1, the AuNPs–ssDNA complex was separated from the dsDNA–AuNPs structure. The remaining complex had a 7 nm distance between FITC and other AuNPs, which caused signal amplification with MEF. BRCA-1 was detected as low as 0.34 fM within 30 min. The utilization of CRISPR-Cas9 eliminated the requirement for nucleic acid amplification, and also presented high sensitivity.

Recently, a hybrid antibody–aptamer integrated immunosensor and close-packed, honeycomb-structured AuNPs nanoarrays were combined for metal-enhanced fluorescent detection of Plasmodium falciparum lactate dehydrogenase (PfLDH), a malaria marker, from whole blood [72]. The target was sandwiched between an oriented malaria antibody and a cyanine 5 (Cy5) modified malaria aptamer for adjusting the distance between the fluorophore molecule and plasmonic nanoarray (10 nm) for optimized plasmonic (Figure 4D). The assay detected malaria target concentrations down to 18 fM (0.6 pg·mL^−1^).

### 3.3. SPR-Based Aptasensors

Since surface plasmon resonance (SPR) biosensors are the most extensively studied class of optical biosensors, they have recently attracted immense interest from the scientific community [132,154,155,156,157,158,159,160]. Research in plasmonics has primarily concentrated on automation, the integration of SPR biosensors, and the development of complex optical transducers based on metallic nanostructures (i.e., nanoplasmonics), which improve the sensing capabilities and facilitate its miniaturization [161,162]. This research has been motivated by an unmet need, hoping that POC biosensors might improve and promote healthcare globally. The research and improvement of surface biofunctionalization techniques have also been crucial to their successful clinical application, improving the sensitivity and selectivity required for a reliable label-free analysis. SPR and LSPR biosensors’ ease of use, reliability, and adaptability have stimulated the development of novel biomedical tests that allow for noninvasive, more precise, timely, and informative detection of human diseases [163]. Considering the basics of SPR-type systems, there are many efforts for adapting, designing, and improving the Kretschmann configuration, which serves as the foundation for the construction of numerous prism-assisted biosensors [164]. To detect biomolecular interactions taking place at the sensor surface, prism-coupled systems use the SPR phenomenon, which manifests as an intensity dip in the reflected light [163]. SPR is a potent and popular biological and chemical sensing technology that can follow molecule interactions in real time [165].

Aptamer-based SPR biosensors have drawn significant attention among the various SPR-based sensing applications due to their ease-of-use, viability, and affordability for target detection [166]. Notably, because there is no space for the aptamer to bind with another molecule, most tiny molecules only bind aptamers with the one-site binding configuration [167]. The mass of the binding component causes variations in the index of refraction at the biosensor surface, which are detectable using an SPR instrument [168].

Over the years, aptamer-based POC urinary biosensors have been established to detect diverse urinary markers at low concentrations, including 8-OHdG [169], cocaine [170], advanced glycation end products (AGEs) [171], and dopamine [172]. A metabolic illness that causes hyperglycemia due to inadequate insulin production, diabetes is a complex chronic disease. To manage blood sugar, one must inject insulin every day for the rest of one’s life. A crucial diagnostic tool for identifying and controlling medical disorders is glycosylated hemoglobin (HBA1C) which is a crucial parameter for determining blood glucose levels [164]. An integrated microfluidic biosensor for testing HBA1C was created by Chang et al. [173]. The technique employed nucleic-acid aptamers to detect HBA1C with high sensitivity and high specificity. The technology reduced the risk of diabetic complications by returning results in 25 min and costing less than conventional procedures. According to Duanghathaipornsuk et al., the performance of the sensing was also influenced by the strength of the binding affinity. For the SPR sensing of hemoglobin and glycated hemoglobin, DNA nanocages were created to increase the binding stability and strength of the aptamers to their target proteins [174]. The DNA aptamer-embedded origami cage structure produced 22-fold and 9-fold increases in binding affinity and selectivity for glycated hemoglobin, compared to the ssDNA aptamer. To detect human thrombin and vascular endothelial growth factor (VEGF) proteins, Chen et al. built a four-chambered microfluidic SPR biosensor based on microarrays of RNA aptamers [175]. The surface transcription reaction of T7 RNA polymerase could directly and swiftly synthesize RNA aptamers in the microfluidic format, enabling one-step multiplexed protein biosensing. Numerous target molecules could be analyzed simultaneously using a single SPR biosensor. The reproducibility of the sensor array had a big impact on how reliable SPR biosensors were. Inoue et al. reported an SPR aptasensor utilizing an inkjet spotter that could accurately regulate the position and volume of an ejected aptamer solution to overcome this issue [87]. Simultaneous observations of SPR signals resulting from various thrombin concentrations were obtained using a portable multianalysis SPR aptasensor with a capillary-driven flow chip. By eliminating manual intervention during the preparation process and utilizing the BlockAce reagent (which was frequently used as a blocking solution with ELISA technology for separating biomolecule spots), this method dramatically increased the reproducibility of SPR aptasensors. Thus, the SPR aptasensor’s detection limit was comparable to that of other SPR biosensors (1 nM). According to Dejeu et al., the analyte recognition caused by the conformational shift of aptamers caused the negative SPR signals to be seen during the detection of tyrosinase [176]. They discovered that aptamer configuration rearrangement caused the refractive index to increase by a small molecule, and the aptamer complex to deviate from the total of the refractive index increments of the constituent parts. These findings offer new perspectives and suggestions for comprehending the consequences of the refractive index increment’s nonlinearity on variations in SPR signal. In addition, Hu et al., have created SPR biosensors for dopamine detection based on the idea of noncovalent aptamer immobilization by covering the surface of a gold film with a single layer of graphene [88]. They demonstrated that the presence of dopamine altered the structure of the aptamer, which was capable of amplifying surface refractive index signals at the fiber surface. It was shown that the use of graphene as a sensing layer for SPR could be useful for small-molecule detection. With a lower limit of detection of 10^−13^ M, the aptasensor displayed remarkable sensitivity.

Sandwich-like detection techniques for aptamers have also been developed, and they are similar to the idea of ELISA [177]. The SPR plastic optical fiber (POF) system with an aptamer-based surface has been created for SARS-CoV-2 spike glycoprotein detection [89]. After creating a mixed layer of gold using a mixture of PEGthiol and BiotinPEGlipo, a streptavidin coating was applied. Then, a biotin-modified aptamer was immobilized on the streptavidin coating. An optical POC device beneficial in the early detection of the SARS-CoV-2 virus, the plastic OF aptasensor has a LOD of 36.7 nM. Previously, VEGF, (chosen as a circulating protein due to its being likely linked to cancer) had been detected in the nanomolar range using an SPR POF system by Cennamo et al. [178]. They had immobilized a DNA-aptamer sequence that is unique to VEGF on the gold surface.

**Figure 5:**
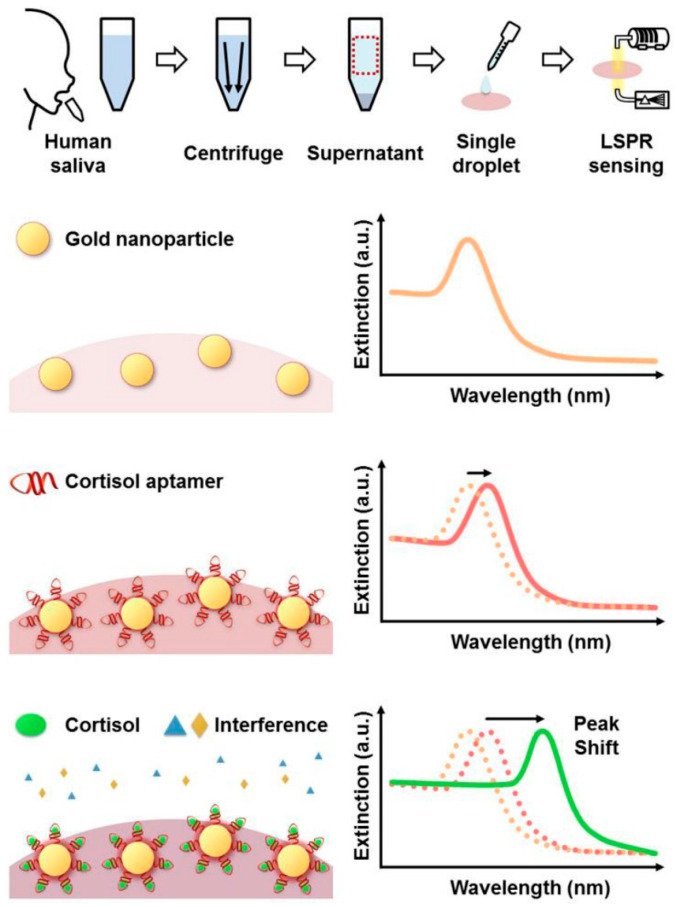
The representation of the procedures needed to sample saliva cortisol and detect salivary cortisol using an LSPR aptasensor [91].

It is well known that the SPR signal can be noticeably amplified with electronic coupling between the localized surface plasmons of AuNPs and the SP waves connected to a gold chip [179,180]. LSPR has an impact on the optical characteristics of AuNPs, which can be exploited to boost SPR sensing [166]. Using the stable chemical conjugation of mercapto- and amino- functional groups to gold, it is simple to link biological ligands with AuNPs. An effective aptamer-based SPR biosensor for breast cancer-derived exosomes with dual gold nanoparticle-assisted signal amplification was established by Wang’s research group [90]. Exosomes collided with the gold substrate that had a CD63 aptamer immobilized on it. To create a sandwich combination of CD63 aptamer/exosome/aptamer–T30–AuNPs, aptamer-coated T30-linked AuNPs (aptamer–T30–AuNP) were then added. This resulted in a single AuNPs-amplified SPR response. To achieve dual-signal amplification, the A30-coated AuNPs were ultimately inserted through the hybridization of two complementary sequences (T30 and A30). This technique made it possible to catch exosomes with a LOD of 5 × 10^3^ exosomes·mL^−1^. The same team recently unveiled a different amplification technique utilizing AuNPs with polydopamine functionalization [181]. On the polydopamine-modified AuNPs, chloroauric acid was reduced with polydopamine molecules to produce tiny AuNPs, which further improved the SPR response. Utilizing polydopamine-modified AuNPs made exosome detection easier than using the earlier technique of poly(A) and T-DNA hybridization. Jo et al. developed a highly sensitive LSPR aptasensor for the direct detection of cortisol in saliva [91]. With a LOD of 0.1 nM, the LSPR aptasensor demonstrated excellent detection performance for a broad range of cortisol concentrations ranging from 0.1–1000 nM. (Figure 5) shows the foundations of sampling salivary cortisol and creating an easy-to-use detection method for the LSPR aptasensor. They showed that using aptamers in LSPR may enhance the detection of small compounds, such as cortisol, when the size of AuNPs and the immobilization of the aptamer were adjusted.

**Figure 6:**
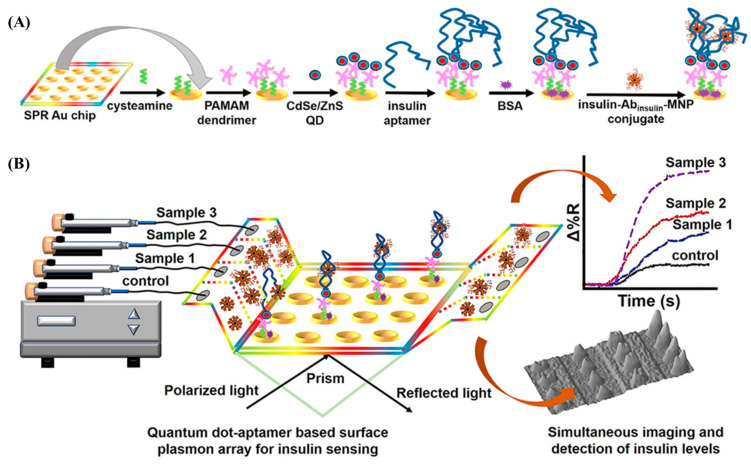
SPR microarray aptasensor design for serum insulin detection. (**A**) The schematic for the aptamer-functionalized QD-based sensor. (**B**) Difference image and respective sensograms of serum insulin-Abinsulin-MNP conjugates binding on the immobilized aptamer microarray [92].

Recently, Singh demonstrated a QD-based aptasensor with aptamer functionality, which was used to detect insulin in diabetes patients’ serum samples [92]. The schematic for the aptamer-functionalized QD-based aptasensor for detecting serum insulin in patient samples is shown in (Figure 6A). High-molecular-weight dendrimers were immobilized on the cysteamine layer with less nonspecific binding. The developed aptasensor was capable of detecting serum insulin concentrations as low as 5 pM, which is critical for identifying insulin levels in challenging clinical samples. The resulting amino groups of cysteamine and the PAMAM dendrimer, to which the carboxylated CdSe/ZnS QDs were bound, were joined by glutaraldehyde. The developed SPR aptasensor’s schematics are shown in (Figure 6B). Because it successfully assessed insulin levels in patient samples with good sensitivity, specificity, and repeatability, the developed QD-plasmon-connected microfluidic aptasensor is favorable.

**Figure 7:**
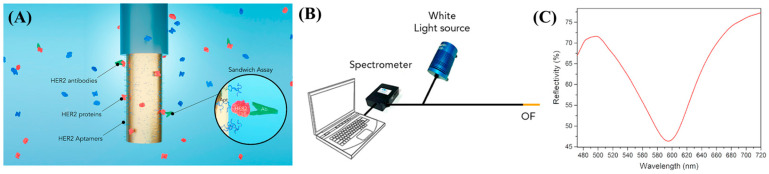
(**A**) The representation of the gold-coated, unclad fiber utilized in a sandwich configuration with amplification from antibodies to detect HER2 molecules using SPR. (**B**) The spectrometer and a white light source are both linked to the optical fiber probe. The instrument may be connected to a laptop and is portable. (**C**) Gaussian SPR curve achieved by the gold-coated OF [93].

Using a HER2 protein biomarker, an OF-SPR for breast cancer detection was published by Loyez et al. [93]. To specifically detect HER2 proteins, anti-HER2 ssDNA aptamers were directly bonded to the gold surface. To target HER2, thiolated aptamers were immobilized on the surface of gold. Anti-HER2 antibodies (20 g·mL^−1^) were used as signal enhancers to lower the device’s limit of detection after the identification of HER2 at low concentrations (Figure 7A). The amplitude spectrum that was released from the OF-SPR setup resulted in a dip-curve, as shown in (Figure 7B,C). Determination of low molecular mass targets on OF-SPR is a challenging task. A single-step cis-duplexed aptamer (cis-DA) and an AuNP-integrated OF-SPR platform were developed for solving this challenge [182]. The target was chosen as ssDNA for proof-of-concept studies. AuNPs were functionalized with aptamer complementary elements (ACE) to create a complex with linker DNA and aptamer conjugation. Before the target’s introduction, AuNPs were found near the surface of gold-coated OF-SPR. The application of target-induced ACE breakage was due to the high affinity of aptamers towards ssDNA. The increasing distance between AuNPs and the fiber surface caused a decrease in the SPR signal in conjunction with the increasing ssDNA concentration. This OF-SPR-based biomarker capture strategy enabled six times the signal amplification compared to cis-DA complex free platform and detected the low molecular mass target, ssDNA, as low as 230 nM.

### 3.4. Electrochemical Aptasensors

Electrochemical methods are advantageous because of their high sensitivity, low detection limit, minimal time consumption, and low cost of equipment [183]. They have received significant attention in developing aptamer-based biosensors due to their low-cost, high sensitivity and selectivity, low detection limits, and the potential to develop flexible POC systems [184,185,186,187].

Recently, significant attempts have been made to develop POC-based electrochemical aptasensors for several biomarkers. An electrochemical aptasensor, for instance, was developed to detect a protein called MPT64 secreted from *Mycobacterium tuberculosis* (a Tuberculosis (TB) causative agent) and these biomarkers were detected using Electrochemical Impedance Spectroscopy (EIS). Briefly, HS-(CH_2_)_6_-OP(O)_2_O-(CH_2_CH_2_O)_6_-TTTTT-aptamer was immobilized on an Au electrode, where Au-based electrodes were used to self-assemble thiol-based aptamers using gold-sulfur (Au-S) bonds. 6-Mercapto-1-hexanol and triethylene glycol mono-11-mercaptoundecyl ether were used as the antifouling agent to avoid nonspecific binding. The aptasensor exhibited a detection limit of 81 pM. It significantly decreased the detection time down to 30 min compared to the detection time of both traditional sputum microscopy and PCR (several hours or even days) [74]. Another electrochemical aptasensor was developed using the same Au–S interaction strategy with a screen-printed gold electrode to detect the Yersinia adhesin A (YadA) biomarker of *Yersinia enterocolitica* from Yersinia species, a species capable of causing diarrhea, mesenteric lymphadenitis, arthritis, and bacterial blood poisoning called sepsis [75,188]. The aptamer was selected using cell-SELEX, and likewise, the immobilization was performed through the Au–S method on a screen-printed gold electrode. The device had a detection limit of 7.0 × 10^4^ CFU·mL^−1^ and could exhibit a linear performance range of 7.0 × 10^4^ CFU·mL^−1^ to 7.0 × 10^7^ CFU·mL^−1^ [75].

**Figure 8:**
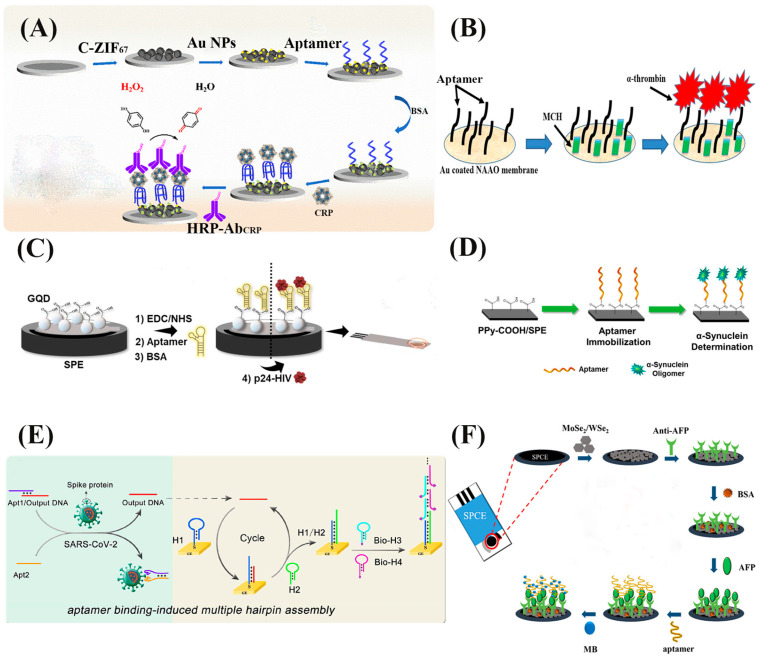
(**A**) Fabrication of the aptasensor for CRP detection with a triple-step signal amplification strategy [76]. (**B**) Fabrication and α-thrombin detection using a Au-coated NAAO membrane aptasensor [79]. (**C**) Aptasensor fabrication for the detection of p24-HIV [81]. (**D**) Fabrication and α-Syn detection of PPy-COOH conductive polymer-based aptasensor [82]. (**E**) Signal-enhancing multiple hairpin assembly for SARS-CoV-2 detection [84]. (**F**) Aptasensor fabrication with 2D MoSe_2_/WSe_2_ and AFP detection [85].

Metal–organic frameworks (MOFs) are self-assembled organic-inorganic crystalline nanomaterials consisting of metal ions surrounded by organic linkers [189]. MOFs have a large surface area that makes them capable of adsorbing functional materials (such as enzymes, DNA probes, and nanoparticles); the high porosity of MOFs enables them to encapsulate signal molecules and release them to generate a strong signal response when in contact with target materials. Moreover, they possess tunability of the framework, which shows excellent potential in the field of biosensor development [190,191,192]. The use of an electrochemical aptasensor with Zirconium-based MOFs (Zr-MOFs), as well as an aptamer as the recognition element for exosomes, exhibited a detection range of 1.7 × 10^4^ to 3.4 × 10^8^ particles·mL^−1^. It could achieve a detection limit of 5 × 10^3^ particles·mL^−1^ [77]. C-reactive protein (CRP) is a nonspecific biomarker for cerebrovascular diseases, myocardial infectious inflammation, and cancer; however, the precise measurement of CRP is very challenging in POC settings [193,194]. An aptamer-based electrochemical biosensor with enhanced signal modality was developed to detect (CRP) using a triple-step strategy. Briefly, the probe molecule number was increased using a horseradish peroxidase-labeled CRP antibody (HRP-Ab_CRP_) enzyme-catalyzed reaction; likewise, the use of a Zeolitic imidazolate framework (ZIF_67_)-MOF with rhomboid dodecahedra structure was observed to improve the specific surface area; and, carbonizing the ZIF_67_ (C-ZIF_67_) increased the conductivity. This three-step strategy enhanced the signal response of the biosensor (Figure 8A). The developed biosensor could achieve a linear response range of 10 pg·mL^−1^ to 10 μg·mL^−1^ and a detection limit of 0.44 pg·mL^−1^ [76].

Thrombin is used as the biomarker for hematological system disorders [78]. Human α-thrombin detecting aptasensor was developed based on the Au–S immobilization method with MCH as the co-adsorbent (Figure 8B) [79]. The Au was coated with either nanoporous anodized alumina or aluminum oxide (NAAO), due to its high surface-to-volume ratio, structured pores, and high pore density [195]. The aptasensor exhibited selectivity towards α-thrombin over γ-thrombin and lysozyme. It could achieve a detection limit of 10 pM with 500 μM concentration of human serum albumin (HSA) interfering protein, which was better than the aptamer functionalized MoS_2_ nanosheet/platinum (Pt) electrode-based thrombin aptasensor (53 pM in 1% human serum) and then the other Au–S binding-based aptasensors as well [196,197]. Furthermore, the implemented 4-electrode system improved the sensor’s accuracy by eliminating the effect of the current carrying outer electrodes over the voltage-measuring electrodes [79].

Materials such as graphene oxide (GO) and graphene have been reported to improve the limit of detection and the sensitivity of electrochemical biosensors due to their large aspect ratio and highly porous structure, respectively. A capacitive biosensor with a laser-induced graphene (LIG) electrode was developed to detect thrombin using the amide group generating 1-(3-dimethylaminopropyl)-3-ethylcarbodiimide hydrochloride (EDC)/N-hydroxy succinimide (NHS) aptamer immobilization strategy [78]. In this method, electrodes were modified with EDC/NHS mixture solution to develop carboxylate group (-COOH) on the electrode. Consequently, the carboxylate group of the electrode could be used to immobilize aptamers modified with amino group (-NH_2_) by creating the covalent amide group (-CO-NH-) [198,199,200,201,202]. The amide bond-based immobilization has much higher stability with no significant loss in signal after being in storage for five days, compared to the 23–30% loss of thiolated DNA/mercaptohexanol electrode-based aptasensors [203,204]. In another study, GO has been used as an enhancer in a voltammetry-based electrochemical aptasensor for thrombin detection, using the same EDC/NHS aptamer immobilization strategy. The aptasensor using GO as an enhancer had a range of 0.005 nM to 50 nM with a detection limit of 1 pM [80]. However, the LIG-based aptasensor could achieve a dynamic range of 0.01–1000 nM and a detection limit of 0.12 pM, which was much higher than the GO enhancer-based aptasensor [78]. A screen-printed graphene quantum dot (GQD) electrode-based electrochemical aptasensor with EDC/NHS immobilized aptamers was developed for the early diagnosis of Human Immunodeficiency Virus (HIV) through the detection of p24-HIV protein, which exhibited linear performance from 0.93 ng·mL^−1^ to 93 μg·mL^−1^, and had a detection limit of 51.7 pg·mL^−1^ (Figure 8C) [81]. The use of polypyrrole-2-carboxylic acid (PPy-COOH) has also been reported to immobilize NH_2_-modified aptamers through amide group (-CO-NH-) generation (Figure 8D). For detecting α-Synuclein (α-Syn), the biomarker for Parkinson’s disease, this PPy-COOH conductive polymer-based aptasensor exhibited linear performance in a range of 1 × 10^–8^ to 0.1 nM and had a detection limit of 1 × 10^–6^ pM [82].

A DNA tetrahedron (TDN) linked dual-aptamer (AS1411 and MUC1) based aptasensor to detect the MCF-7 breast cancer cells was developed on Au electrodes based on the Au–S approach and modified with MCH, Pt nanoparticles decorated porous coordination network-224 (PCN-224), G-quadruplex/hemin DNAzyme (GQH), and horseradish peroxidase (HRP). The use of TDN provided the sensor with a uniform interface, using two aptamers increased the cell culture efficiency, and using Pt/PCN-224 enhanced the signal. Moreover, GQH and HRP increased the catalytic activity, thus improving the aptasensor’s overall performance. The sensor exhibited a linear response range of 20 to 1 × 10^7^ cells·mL^−1^ and a detection limit of 6 cells·mL^−1^ [83]. Using dual aptamers has also been reported in the development of a sensor for odontogenic ameloblast-associated protein (ODAM), a periodontal disease biomarker in gingival crevicular fluid (GCF) [205].

Avidin or streptavidin are tetrameric proteins with an affinity for biotin that have been reported to be used to immobilize biotin-labeled aptamers. The streptavidin–biotin immobilization was used to develop a dual-aptamer POC electrochemical aptasensor with an enhanced signal response for detecting SARS-CoV-2, the causative agent of COVID-19. Two aptamers bound the SARS-CoV-2 target in a sandwich assay for proximity binding, increasing the local concentrations of DNA strands. This increased concentration resulted in the displacement of DNA, converting the SARS-CoV-2 to output DNA that induced a chain reaction of hybridization, creating long and linear concatemers with multiple hairpin assemblies. These multiple hairpin assemblies were labeled with biotin, and immobilized a substantially large amount of streptavidin–alkaline phosphatase (ST–ALP). This large quantity of ALP could generate an enhanced signal reaction when reacting with 1-naphthol phosphate (1-NPP) through an electrochemical oxidation reaction (Figure 8E). The sensor exhibited a linear range from 50 fg·mL^−1^ to 50 ng·mL^−1^ and a detection limit of 9.79 fg·mL^−1^ [84]. Two-dimensional (2D) materials have been reported to improve the electrode reactivity and chemical loading quantity on the electrode of biosensors due to their electrochemical properties and high active surface area [206]. Hence, an electrochemical aptasensor was developed by modifying a screen-printed carbon electrode with 2D MoSe_2_/WSe_2_ to detect a biomarker for primary liver cancer named prime alpha-fetoprotein (AFP); this was able to achieve a detection limit of 0.65 pg·mL^−1^ (Figure 8F) [85]. Moreover, the addition of nanomaterials, such as AuNPs, has also been attempted, and caused an improvement in the signal response; as a result, the detection limit decreased from 24 pg·mL^−1^ to 2.4 pg·mL^−1^ [207].

A reagentless electrochemical biosensing method named nanoscale molecular pendulum (NMP), capable of detecting protein biomarkers for cancer and COVID-19, has been developed by immobilizing antibody-conjugated DNA on a screen-printed electrode (SPE) [208]. Ferrocene was used as the redox reporter in this method that oxidized at an approximate potential of +400 mV. However, when the target was bound by the antibody, the target-bound antibody moved slower, delaying the oxidation, and this change in oxidation was used as the sensing principle [208,209]. The developed SARS-CoV-2 sensor for COVID-19 detection could exhibit a LOD of 1 fg·mL^−1^ for nucleoproteins and 20 copies·mL^−1^ for viral particles [208]. A paper-based electrochemical aptasensor was developed for cancer biomarker detection, and it was capable of simultaneously measuring two biomarkers, carcinoembryonic antigen (CEA) and neuron-specific enolase (NSE), using two sensing electrodes. This paper-based aptasensor offered a low production cost, and could exhibit high sensitivity, with a detection limit of 2 pg·mL^−1^ for CEA and 10 pg·mL^−1^ for NSE. In addition, the aptasensor showed a maximum relative error of 7.81% for CEA and 22.43% for NSE, when compared to commercially available systems. Furthermore, the simultaneous detection of multiple biomarkers showed the potential to improve the performance accuracy of POC testing and achieve the early detection of cancer [86]. Methods such as inkjet printing have also been attempted by developing carbon nanotube (CNT)-aptamer complex ink based on the strong π–π stacking interaction of the nucleotide bases of the ssDNA aptamer and sidewalls of the CNT. The developed aptasensor exhibited a detection limit of 90 ng·mL^−1^ against lysozyme with a linear range of 0 to 1.0 µg·mL^−1^ [210]. Other flexible platforms, such as polyester films, have also been considered for fabricating sensors with more robust structures while keeping them flexible. A screen-printed electrochemical strip for the early detection of Alzheimer’s disease (AD) was developed on a polyester film by detecting the decrease in the amount of miRNA-29a. The decline of miRNA-29a levels in brain has been reported among AD patients, which increases the BACE1 gene expression, itself a risk factor for Alzheimer, making miRNA-29a a promising biomarker for AD. An anti-miRNA-29a probe was used for the specific binding of the anti-miRNA-29a, which was tagged with methylene blue (MB) as the redox mediator to generate the electrochemical signal, and AuNPs were used to improve the signal. The sensor showed a LOD of 0.2 nM in human serum [211].

## 4. Computational Approaches for Aptamer Modeling and POC Testing Integrations

After elaborating aptasensors and their properties, and considering the complexity of the bio–nano interface in their structures, one must inquire as to which simulation and theoretical computation methods can be used to study biosensors in a realistic and meaningful way [212]. Due to experimental limitations at the microscopic level, the integrated approaches that combine in silico design with experimental measurements have immense potential to exacerbate the hurdles involved in developing biosensors [212,213]. This part of the review presents an overview of the application of Molecular Docking calculation, Molecular Dynamics Simulation (MD), Density Functional Theory (DFT), Quantum Mechanics and Molecular Mechanics (QMMM), and Artificial intelligence (AI) to study the typical disease biomarkers, including aptamer, to design and develop biosensors [214].

### 4.1. Molecular Docking Calculation and Molecular Dynamics Simulation

Briefly, MD simulations predict how every atom in an aptamer, protein, or other molecular systems moves over time, in accordance with a general model of physics [215]. A variety of important biomolecular processes, including ligand binding, protein folding, and conformational changes can be captured with these simulations. MD simulations are often applied in combination with experimental structural biology techniques including cryo-electron microscopy, NMR, FRET, Electron Paramagnetic Resonance (EPR), and X-ray crystallography [216]. In particular, aptamers have been studied in silico to enable the identification of high-affinity aptamers for the design and development of biosensors [215]. For instance, the use of molecular docking calculation, MD simulation, and electrochemical measurements identified five sensitive and selective RNA aptamers to apply for designing an electrochemical biosensor to detect ammonium dissolved in water. They validated their work experimentally by detecting target molecules and found that using different aptamers led to a significant difference in the biosensor’s response [217]. Streptomycin is an aminoglycoside antibiotic that is used to treatment of human and animal infections caused by bacteria. Because of the serious side effects of this drug and finding a way to detect its trace amount in food products and serum, Nosrati and Roushani applied molecular docking calculations and MD simulations to predict the binding pocket of 79-mer ssDNA aptamer in the interaction with streptomycin. Their findings were in fair agreement with experimental results and help to optimize aptamer efficiency in biosensing applications [218]. Chen et al. proposed an optical liquid crystal (LC) biosensor (label-free, cost-effective, and aptamer-based) for detection of insulin. They showed that their sensor is able to detect insulin in diluted human urine and serum, and thus has a potential basis for POC testing [219]. Zhao et al., investigated a rapid and sensitive aptamer-based biosensor for the detection of domoic acid (DA), an amnesic shellfish toxin produced by red tide algae known as *Pseudo-nitzschia*. They explored the binding mechanism between DA and the aptamer using a molecular docking calculation and MD simulation. In their study, the time of the process of detection was 7 min [220].

### 4.2. Density Functional Theory

DFT is a mature theory that provides a reliable method for studying materials in their crystal state, as well as their molecular structure. Various commercial and open-source software are available to execute the DFT computations for particular systems. The combination of DFT and the designed experiment would be helpful to further our understanding of how to design efficient, reliable, and cost-effective biosensors [221,222]. In 2020, Ouyang et al. designed a self-powered photoelectrochemical (PEC) aptasensor for ultrasensitive detection of Microcystin-LR (hepatotoxins released by cyanobacteria during eutrophication process that is the most ubiquitous, plentiful, and varied congener) and they used DFT computation to study the electron transfer path of the system. They proved that their structure would be a good candidate in the field of sensing [223]. For the first time, Ouyang et al. synthesized a 3D-printed bionic self-powered sensing device. Experimental results and DFT computation showed that their aptasensor had high sensitivity and selectivity to the detection of Bisphenol A (BPA) environmental toxins and active endocrine disrupters. Their work provides a new strategy to detect BPA in food and environmental samples [224]. Fernandez et al., reported a disposable POC sensing platform specific for the detection of salivary cortisol. Their biosensor used a cortisol-specific aptamer to achieve high specificity to cortisol, and they then analyzed the activity of their biosensor using DFT computation. The potential of their work was demonstrated by detecting the salivary cortisol variations in five humans [225]. Li et al., fabricated the (BiVO4/2D-C3N4/DNA) aptamer photoelectrochemical (PEC) biosensor. Using DFT computation, they showed that their sensor provided an excellent detection property for Microcystin-LR molecule. In addition, by changing the DNA aptamer, their biosensor showed a higher sensitivity for the detection of heavy metal ions, antibiotics, and tumor markers [226]. Using a CD63 aptamer and coupling DNA nanotechnology with single-atom catalysts, Zeng et al., synthesized the single-atom biosensor. Their product served as a photoelectrochemical sensing platform to detect biomolecules. Their study introduces new opportunities for protein diagnostics and biosecurity [227].

### 4.3. Quantum Mechanics and Molecular Mechanics

**Figure 9:**
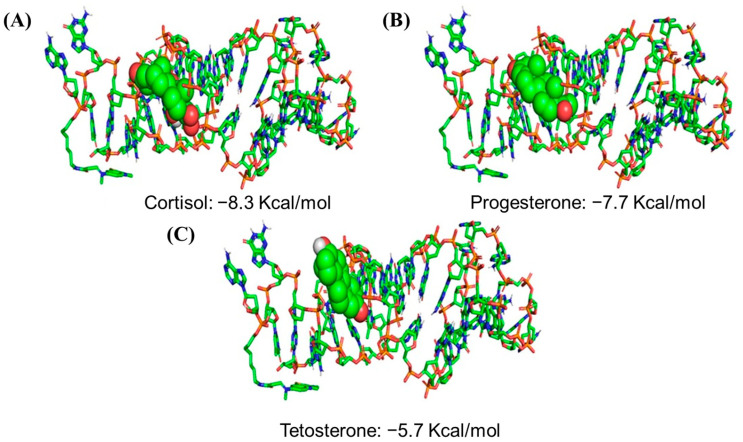
Molecular Docking results of the interaction of aptamer and (**A**) cortisol, (**B**) progesterone, and (**C**) testosterone [230].

Applying the QMMM methodology for studying biological systems containing several thousands of atoms can be a promising strategy due to overcoming the cost of simulation [228]. QMMM theory is an efficient approach to simulate physicochemical phenomena while the modifications of electronic structures can be modeled using quantum mechanical approaches and the environment can be approximated using classical molecular mechanical approaches [229]. A combination of QMMM with the experimental result can provide us with a possible way to study the modified systems. In 2022, for the first time, Karuppaiah et al. reported an electrochemical cortisol aptasensor formed by conjugating methylene blue with cortisol-specific DNA aptamers through the use of QMMM calculations and molecular docking calculations. According to their results (Figure 9), the aptasensor measured clinically meaningful cortisol levels in human serum without any reagent [230]. By using a highly controlled covalent functionalization strategy and QMMM calculation, Purwidyantri et al., could design ultrasensitive aptamer-based biosensors that can early detect hepatitis C virus (HCV) core protein, facilitating the early diagnosis of HCV infections. Their device is specific, and achieves attomolar detection of the viral protein in human blood plasma [231].

### 4.4. Artificial Intelligence (AI)

The application of AI and biosensors has caused the cross-disciplinary concept of AI-biosensors. In usual flexible bioelectronic materials, such as textiles, flexible films, bandages and patches, play a significant role in AI-biosensors [232]. Intelligent hydrogels that are sensitive to pH, temperature, ions, and molecules [233], in addition to carbon materials with such properties as electrical conductivity and light weight flexibility [234], as well as smart polymers that can change color, solubility, or shape when affected by temperature, [235] are some examples of flexible bioelectronic materials that will facilitate the fabrication of AI-biosensors. Furthermore, the AI-biosensors have relevance to wireless data communication, such as radio-frequency identification, Wi-Fi, and Bluetooth, to transmit the information between biosensors and smartphone-based platforms [232]. Herein, it should be mentioned that AI is the extracting knowledge from data without human intervention [236] and this method is suitable for the prediction of massive sequences in comparison to the structured-based methods that are not proper for predicting the affinity of a large number of sequences to one target simultaneously [47].

## 5. Conclusions

**Figure 10:**
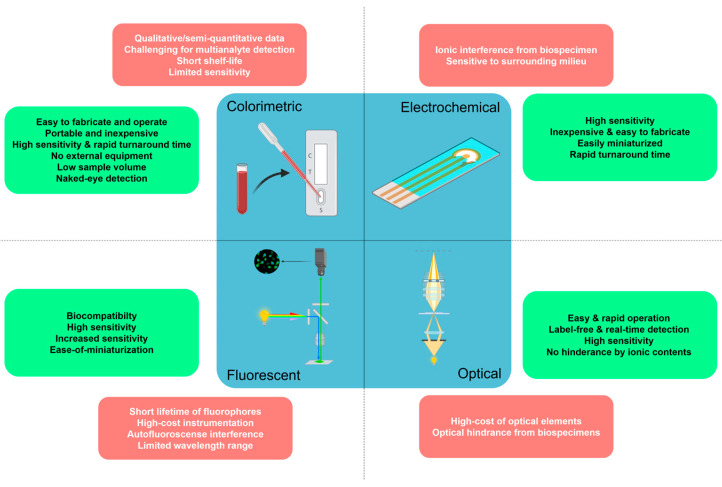
The pros and cons of aptamer-based POC devices. Created with BioRender.com (accessed on 11 May 2023).

Tracking biomarkers in physiological fluids provides us with a wealth of information regarding illness presence, treatment progress, and disease risk. In particular, an ideal POC biosensor holds great potential to provide critical information to healthcare providers and patients in resource-constrained countries. The term ASSURED (affordable, sensitive, specific, user-friendly, rapid, equipment-free, delivered) was coined to define a flawless test for developing countries where resources are limited [13]. For instance, Sinha et al. developed a microfluidic chip biosensor with field-effect transistor sensor arrays, using aptamers as capture probes, for the detection of cardiovascular disease biomarkers [32]. Given that it meets practically all of the ASSURED standards, this chip proved very compatible for diagnostics. The device was portable and automated, minimizing human intervention, and the chip could operate with clinical samples that had not yet been processed. Additionally, the analysis’s sample volume was small, and the detection time was quick. Despite researchers working hard to bring microfluidics to the market, there are very few microfluidic chips commercially available that meet the ASSURED criteria. The market adoption of microfluidic technology, complicated and time-consuming governmental approval procedures, and customer acceptance are barriers to its commercialization [237]. The REASSURED requirements, where R and E stand for real-time connectivity and ease of sample collection, will be the new standards that must be met by next-generation POC diagnostic equipment. As shown in (Figure 10), fluorescent and electrochemical POC sensors are highly sensitive, and their shelf life and stability are limited. Colorimetric POC sensors are easily available, low-cost, and sensitive, but have a short shelf-life. On the other hand, SPR POC sensors are portable, easy to operate and provide real-time analysis, but they are expensive and require advanced devices.

Antibodies have traditionally been used as the primary recognition element in biosensing studies, but aptamers are becoming an alternative choice due to their high affinity. Aptamers possess several advantages over antibodies, such as their ability to recover their native conformation with slight changes in pH, salt concentration, chelating agents, and temperature. Antibodies, in contrast, can undergo irreversible denaturation when exposed to these conditions, resulting in a permanent loss of their biological activity and binding ability. Aptamers can be synthesized in large quantities with high accuracy, reproducibility, and at a lower cost than monoclonal antibodies, which are laborious and expensive to produce. Unlike modified antibodies with a loss of binding activity, aptamers can undergo chemical modifications without sacrificing their binding affinity. Aptamers also have excellent thermal stability, which allows them to withstand room temperature conditions for extended periods without loss in their activity. In addition, aptamers are nontoxic and have low immunogenicity, making them suitable for both in vitro and in vivo applications. Before aptamers can be employed in clinical trials or made commercially available, more needs to be learned about their pharmacokinetics and interactions with their targets, as the research into them is still in its infancy. In this review, we have elaborated the usage of aptamers for designing and developing novel POC biosensors, such as electrochemical, optical, colorimetric, and fluorescent. Between these biosensors, most studies have been carried out through optical modality including colorimetric, fluorescent, and SPR, and followingly, electrochemical biosensors account for the second greatest number of efforts in this area. In addition, we have mentioned the studies that use molecular docking, MD simulation, DFT, and QMMM computation, in addition to the role and application of AI for tackling the experimental limitations of aptamer development. It should be mentioned that the number of studies that used the QMMM computations was low, and increasing the number of studies on these computations can be a possible development for future studies.

## Figures and Tables

**Table 1:** Evaluating POC devices in terms of the target analyte, physical condition, sensing principle, and limit of detection (LOD).

Target Analyte	Physical Condition	Sensing Principle	Limit of Detection (LOD)	Reference
Cortisol	Stress	Colorimetric	0.37 ng·mL^−1^	[53]
Dopamine	Alzheimer’s, Parkinson’s, and Huntington’s diseases	Colorimetric	10 ng·mL^−1^	[54]
Glycated albumin	GDM	Colorimetric	0.8 mg·mL^−1^ and 1.5 mg·mL^−1^	[55]
PDGF-BB and thrombin	Tumor regions (e.g., liver, gastrointestinal tract) and hemostasis	Colorimetric	1.0 nM and 1.5 nM	[56]
CXCL 9	Antibody-mediated rejection of kidney transplantation	Colorimetric	10 pg·mL^−1^	[57]
K^+^	Chronic kidney disease	Colorimetric	0.01 mM	[58]
HER2	Breast cancer	Colorimetric	10 nM	[59]
RBP4	Type 2 diabetes mellitus	Colorimetric	90.76 ± 2.81 nM	[60]
Exosomes	Leukemia	Colorimetric	42 particles·μL^−1^	[61]
IL-6	Brain injury or inflammation	Colorimetric	1.95 μg·mL^−1^	[62]
Mycobacterium tuberculosis DNA	TB	Colorimetric	0.28 nM	[63]
PDGF-BB	Tumor growth and progression	Colorimetric	10 fM	[64]
Cortisol	Stress	Fluorescent	1 nM	[65]
Aβ and tau protein	Alzheimer	Fluorescent	50 pM and 10 pM	[66]
Mucin 1	Tumor	Fluorescent	0.15 fg·mL^−1^ mucin 1 or 3 CTCs·mL^−1^	[67]
AFP	Hepatocellular carcinoma	Fluorescent	400 pg·mL^−1^	[68]
Mutated BRCA-1	Breast cancer	Fluorescent	0.34 fM	[69]
Cortisol	Stress	Fluorescent	6.76 ng·mL^−1^	[70]
Ig E	Allergic disease	Fluorescent	0.13 IU·mL^−1^	[71]
PFLDH	Malaria	Fluorescent	18 fM (0.6 pg·mL^−1^)	[72]
Glucose, ATP, L-Tyrosinamide, and thrombin	Diabetes, molecular marker for cellular energy, metabolic syndrome and melanoma, and hemostasis	Fluorescent	1.1 mM, 0.1 mM, 3.5 µM and 25 nM	[73]
MPT64 secreted from Mycobacterium tuberculosis	TB	Electrochemical	81 pM	[74]
YadA	Diarrhea, mesenteric lymphadenitis, arthritis, and sepsis	Electrochemical	7.0 × 10^4^ CFU·mL^−1^	[75]
CRP	Cerebrovascular diseases, myocardial infectious inflammation, and cancer	Electrochemical	0.44 pg·mL^−1^	[76]
Exosomes	Cancer	Electrochemical	5 × 10^3^ particles·mL^−1^	[77]
Thrombin	Hemostasis	Electrochemical	0.12 pM	[78]
α-thrombin	Blood clotting cascade	Electrochemical	10 pM	[79]
Thrombin	Anticoagulation and cardiovascular disease	Electrochemical	1 pM	[80]
p24-HIV protein	HIV	Electrochemical	51.7 pg·mL^−1^	[81]
α-Syn	Parkinson’s disease	Electrochemical	1 × 10^–6^ pM	[82]
MCF-7 breast cancer cells	Breast cancer	Electrochemical	6 cells·mL^−1^	[83]
SARS-CoV-2	COVID-19	Electrochemical	9.79 fg·mL^−1^	[84]
AFP	Liver cancer	Electrochemical	0.65 pg·mL^−1^	[85]
CEA and NSE	Cancer	Electrochemical	2 pg·mL^−1^ and 10 pg·mL^−1^	[86]
Thrombin	Blood coagulation cascade	SPR	1 nM	[87]
Dopamine	Neurological and psychiatric disorders	SPR	10−13 M	[88]
SARS-CoV-2 spike glycoprotein	COVID-19	SPR	36.7 nM	[89]
Exosomes	Breast cancer	SPR	5 × 10^3^ exosomes·mL^−1^	[90]
Cortisol	Stress	LSPR	0.1 nM	[91]
Insulin	Diabetes	SPR	5 pM	[92]
HER2 proteins	Breast cancer	OF-SPR	20 g·mL^−1^	[93]

Abbreviations: CXCL 9: CXC-motif chemokine ligand 9, PDGF-BB: Platelet-derived growth factor-BB, HER2: Human epidermal growth factor receptor 2, RBP4: Retinol-binding protein 4, IL-6: Interleukin-6, Aβ: Amyloid beta oligomer, AFP: Alpha-fetoprotein, BRCA-1: Breast cancer gene-1, Ig E: Immunoglobulin E, PFLDH: Plasmodium falciparum lactate dehydrogenase, ATP: adenosine triphosphate, YadA: Yersinia adhesin A, CRP: C-reactive protein, α-Syn: α-Synuclein, CEA: Carcinoembryonic antigen, NSE: neuron-specific enolase, OF-SPR: optical fiber-surface plasmon resonance, K+: Blood potassium ion, HIV: Human immunodeficiency virus, AFP: Prime alpha-fetoprotein/alpha-fetoprotein, TB: Tuberculosis, CTC: Circulating tumor cells, GDM: Gestational diabetes mellitus, CFU: Colony-forming unit.

## Data Availability

Not applicable.
